# Adverse drug reactions in inpatients of internal medicine wards at a tertiary care hospital: A prospective cohort study

**DOI:** 10.4103/0976-500X.77102

**Published:** 2011

**Authors:** Mukeshkumar B. Vora, Hiren R. Trivedi, Bharatbhai K. Shah, C. B. Tripathi

**Affiliations:** *Department of Pharmacology, Government Medical College, Bhavnagar, India*; 1 *Department of Pharmacology, Shri Meghji Pethraji Shah Medical College, Jamnagar, India*; 2 *College of Dental Science and Research Institute, Ahmedabad, Gujarat, India*

**Keywords:** Adverse drug reaction, adverse drug reactions, cohort study

## Abstract

**Objective:**

To find out incidence of adverse drug reactions (ADR) in patients of internal medicine wards and study various aspects of ADR, e.g., causality, mortality, drugs commonly causing ADR in internal medicine wards of Guru Gobind Singh Hospital, Jamnagar, a tertiary care hospital.

**Materials and Methods:**

This was prospective, observational study carried out at Department of Medicine, Shri Meghji Pethraj Shah Medical College attached with Guru Gobind Singh Hospital, a tertiary care teaching hospital, Jamnagar, Gujarat over a period of 6 months. For statistical analysis, ADR were analyzed by using Chi-square test.

**Results:**

Out of total 860 patients admitted, 830 were analyzed as they met the inclusion criteria. A total of 45 (5.42%) patients developed 47 ADR. Among them, 27 (3.25 %) (95% CI, 2.03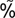, 4.47%) patients due to ADR required hospital admission in medicine ward (ADR Ad), 18 (2.17%) (95% CI, 1.17%–3.17%) patients developed ADR while already hospitalized in medicine ward (ADR In). Most of the fatal and life-threatening reactions occurred due to chemotherapeutic agents. Majority of patients discontinued suspected drug and recovered from ADR.

**Conclusion:**

Fatal and life-threatening adverse reactions reported in the present as well as other studies underline the importance of such studies and need for creating awareness among health professionals about looking for and reporting such reactions.

## INTRODUCTION

Drugs are double edged weapons; they can save life but also can cause adverse drug reactions (ADR). ADR are a major cause of morbidity and mortality worldwide.[1–4] According to World Health Organization (WHO) an ADR is defined as “A noxious, unintended and undesirable effect that occurs as a result of dose normally used in man for diagnosis, prophylaxis and treatment of disease or modification of physiological function.”[5]

ADR in hospital patients can be divided into two broad categories: those that cause admission to hospital, and those that occur in in-patients after hospital admission. Approximately 5% (range 2%–20%) of reported hospitalizations are because of an ADR,[6] and at least one ADR has been reported to occur in 10%–20% of hospitalized patients.[7] They require expensive emergency room care. An ADR is associated with a significantly prolonged length of stay, increased economic burden, and almost twofold increased risk of death.[8] It is fourth to sixth leading cause of mortality in the United States of America.[3]

An ADR reporting program on an institutional basis can support the setting up of a sound pharmacovigilance system in the country. Furthermore, productive hospital-based ADR program can provide valuable information about potential problems in drug usage.[9] ADR occurring in hospital setting can be attributed to severity and complexity of disease process, use of multiple drugs, drug interactions, and possible negligence.[8] ADR could be monitored through active participation or through voluntary reporting system in hospital set up. The objective of this study were to find out incidence of ADR in patients of medicine wards and study various aspects of ADR, e.g., causality, mortality, drugs commonly causing ADR in internal medicine wards of Guru Gobind Singh Hospital, Jamnagar, a tertiary care hospital.

## MATERIALS AND METHODS

This was a prospective observational cohort study conducted at the Department of medicine, Shri Meghji Pethraj Shah Medical College and associated Guru Gobind Singh hospital, Jamnagar, Gujarat (India). This study was conducted for period of 6 months in a cohort of hospital patients. All patient related information was collected as per case record form. Any untoward event was labeled as ADR only after discussing with the treating physician. In the case of any difference of opinion with respect to reaction, treating physician’s opinion was considered as final. In case of doubt investigation was carried out with consent of physician to confirm ADR. No intervention was made in the treatment; observations, remarks, diagnosis, or management of the patient’s disease. All treating physicians and patients were assured regarding confidentiality of the patient information. The patients meeting the following criteria were included in the study:

1. All patients of either sex and age >12 years admitted in internal medicine wards.
2. Patients referred to higher center, or discharged against medical advice but in whom the outcome of ADR was known.
3. Patients transferred from Intensive Care Unit and Intensive Coronary Care Unit to internal medicine wards.

Patients referred to higher center, or discharged against medical advice and in whom outcome of ADR was not known, patients transferred to some other department during his/her drug treatment and those directly admitted to Intensive Care Unit, dialysis unit, TB chest ward, isolation ward were not included in the study. An approximate sample size (*n* = 763) was calculated using a pilot study.[10] Patients were followed up till discharged.

### Statistical analysis

Results are expressed in absolute number and percentages. Comparisons between incidences of ADR in different age groups were performed using Chi-square test. *P* < 0.05 is considered significant.

## RESULTS

A total of 860 patients were admitted in the internal medicine wards during study period and 30 patients were excluded as they did not fulfill the inclusion criteria. All patients included in the study were followed up daily. Drug therapy and any changes made in the same were recorded till the patient was discharged. Of the 830 patients, 45 had developed 47 ADR. Therefore, the incidence of ADR amounts to 5.42% in internal medicine wards. Maximum number of ADR (24 /45) occurred in age group 21–50 years [Table 1]. Total number of patients in this group was 421. The incidences of ADR in males and females were 3.37% and 2.05%, respectively [Table 2].

**Table 1:** Age groups of patients and adverse drug reaction (*n* = 830)

Age groups	Number of patients		
	With ADR	Without ADR	Total
Group 1 (12-20 years)	08 (0.96)	077 (9.28)	085 (10.24)
Group 2 (21-50 years)	24 (2.89)*	397 (47.83)	421 (50.72)
Group 3 (51-65 years)	08 (0.96)	188 (22.65)	196 (23.61)
Group 4 (>65 years)	05 (0.60)	123 (14.82)	128 (15.42)
Total	45 (5.42)	785 (94.58)	830 (100)

Figures in parenthesis denote percentage

* *P* < 0.01 significantly different compared to other age groups.

**Table 2:** Sex of patients and adverse drug reaction (n = 830)

Sex	Number of observed patients		
	With ADR	Without ADR	Total
Male	28 (3.37)	439 (52.89)	467 (56.26)
Female	17 (2.05)	346 (41.69)	363 (43.74)
Total	45 (5.42)	785 (94.58)	830 (100)

Figure in parenthesis denotes percentage.

**Figure 1:**
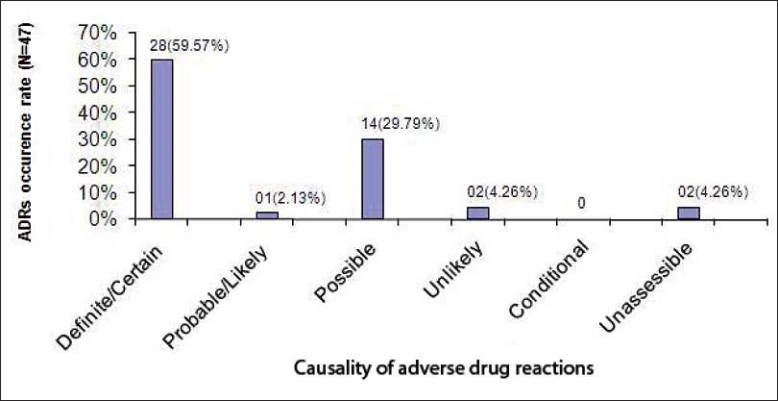
Causality of ADR and its occurrence rate

**Figure 2:**
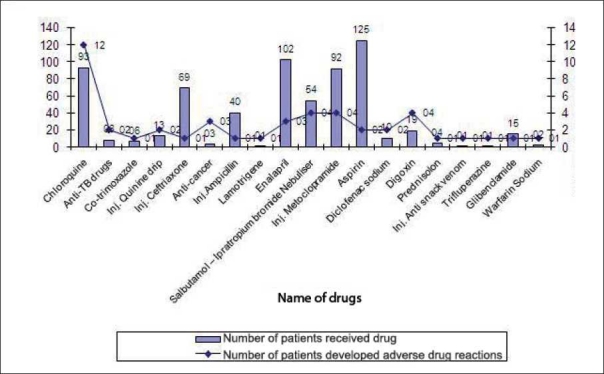
Drugs causing adverse drug reactions

**Figure 3:**
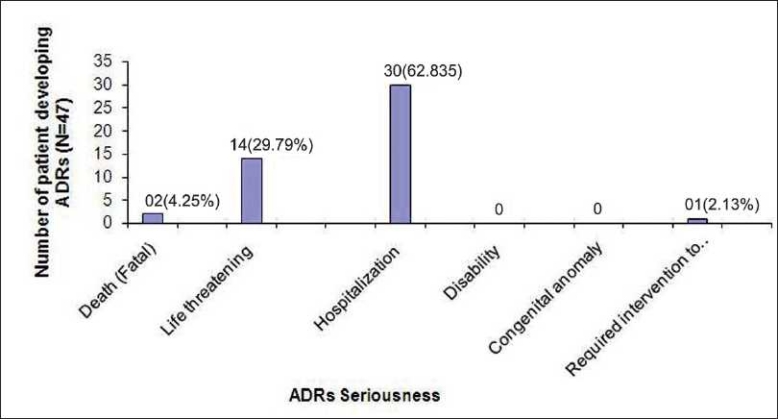
Seriousness of adverse drug reactions.

The causality assessment (World Health Organization causality assessment criteria) of 47 ADR is depicted in Figure 1. Twenty eight (59.57%) ADR classified as “Certain,”; 14 (29.79%) as “Possible.” Among 830 patients studied, 19 groups of drugs were found to cause various ADR. Among these, total 6 drugs from chemotherapeutic group of drugs produced 19 numbers of ADR. Chloroquine phosphate was the most common drug causing ADR [Figure 2]. It was found that 26 (3.13%) patients suffered ADR who received up to 3–5 numbers of drugs, and 16 numbers of patients having ADR received 2 drugs [Table 3]. As far as serious ADR are concerned (as per World Health Organization definition), 2 (4.25%) were fatal, 14 (29.79%) were life-threatening, and 30 (62.83%) required hospitalization (initial or prolonged) [Figure 3]. The management of ADR and outcome are given in tables [Tables 4 and 5, respectively].

**Table 3:** Total number of drugs administered and adverse drug reaction (n = 830)

Number of drugs	No. of observed ADR	No. of observed no ADR	Total
Up to 2	16 (1.93)	111 (13.37)	127 (15.30)
3-5	26 (3.13)**	398 (47.95)	424 (51.08)
6-10	05 (0.60)	270 (32.53)	275 (33.13)
>10	00	004 (0.48)	004 (0.48)
Total	47 (5.66)	783 (94.34)	830 (100)

Figures in parentheses show percentage

* *P*<0.01 significantly different compared to other groups.

**Table 4:** Management of adverse drug reaction (n = 47)

Management of ADR	Total
Continue with suspected drug	02 (4.26)
Discontinue with suspected drug	28 (59.57)
Dose reduced	04 (8.51)
Addition of some other drug	13 (27.66)
Replacement of suspected drug	00
Total	47 (100)

Figure in parenthesis denotes percentage.

**Table 5:** Outcome for management of adverse drug reaction (n = 47)

Outcome of adverse drug reaction	Total
Alive with sequale	0
Recovered	41 (87.23)
Still under treatment	04 (8.51)
Died	02 (4.25)
Total	47 (100)

Figure in parenthesis denotes percentage.

## DISCUSSION

The incidence of ADR in internal medicine wards in this study is higher than other published studies.[11] Sharma *et al*. reported an 4.67% incidence of ADR in a pharmacovigilance study as compared to 2.17% (95% CI, 1.17%–3.17%) observed in in-patients. The percentage of patients requiring admission was 3.1% which was slightly lower than that observed in our study (3.25%; 95% CI, 2.03%–4.47%).[12] Majority of the ADR occurred in the age group of 21–50 year (group 2, *n* = 24). However, maximum number of admissions occurred in this group of patients (*n* = 421) which needs to be kept in mind. This report thus slightly differs from other studies.[13,14]

Males have greater risk of ADR (*n* = 28, 3.37%) than females (*n* = 17, 2.05%). However, this needs to be interpreted in the light of higher number of male admissions. There are various factors affecting the ADR incidence, e.g., age of patients, gender, number of drug exposure, length of hospital stay, genetic factors, ethnicity, dietary, and environmental factors, etc. The main factor affecting differences in ADR incidence could be attributed to inconsistent or contradictory methods among the individual studies. Another example of inconsistent methodology is problem that some investigators include error in administration of drug, overdose of drug for reporting ADR.[7]

In our study antimicrobial-induced ADR were 40.43% which is similar to that reported by Leape *et al*.[15] In these antimicrobial groups, chloroquine phosphate was the most commonly involved drug causing ADR. This suggests the need to increase the awareness with regard to prescription of chloroquine phosphate particularly in patients with suspected malaria. In this study, occurrence of serious ADR (5.42%) seems to be higher compared to that cited in previously published reports.[12] A wide spectrum of life-threatening ADR, including hypersensitivity reactions, acute muscle dystonia, and digoxin toxicity were noticed. It is well-established fact that as the number of drugs increases, the chances of developing ADR also increases. But in present study, patients on 3–5 drugs showed increased ADR but when drugs were more than 10 in number, there was no increase or decreased ADR in the patients.

So far as management regarding ADR is concerned most of the drugs that were thought to cause ADR were discontinued (*n* = 28, 59.57%) and the majority of patients fully recovered (*n* = 41, 87.23%) before discharge from internal medicine wards. Mortality due to ADR was 0.2% of the total admissions, higher than the previous study.[11] The two fatal reactions were observed in the study were related to streptomycin-induced nephrotic syndrome and docetaxel (anticancer drug)-induced immunosuppression. The present study clearly shows that when one looks for occurrence of ADR consciously, more patients with ADR can be picked up and identified having ADR whether physician induced or drug induced. There is a need to inform and educate the treating doctor about the importance of observing for ADR following drugs, recording them correctly and reporting to the concerned authority. This practice will prove to be valuable in making the drug therapy safe and rational. There were some limitations of our study, e.g., small sample size, short period of 6 month duration; and patients are only from general medicine wards.

## CONCLUSION

In conclusion of our study, occurrence of ADR was found in internal medicine wards to be higher, especially that of serious ADR, compared to that reported in previous studies. ADR in hospitalized patients of internal medicine wards were less than those encountered in the patients who got admitted because of ADR. As quite a few reactions happen to be life-threatening/fatal, it is necessary to establish some regular reporting program. This study is useful as a preliminary in initiating a culture of ADR reporting among the health care professionals in hospital.
